# Geriatric Core Curriculum Module Development

**DOI:** 10.1093/geroni/igaf122.739

**Published:** 2025-12-31

**Authors:** Iriana Hammel, Elizabeth Chapman

**Affiliations:** Miami VA Medical Center, Miami, Florida, United States; William S. Middleton Memorial Veterans’ Hospital, Madison, Wisconsin, United States

## Abstract

GRECC ADEEs formed a workgroup to develop an interprofessional geriatric core curriculum using the Geriatric 5Ms (‘AFHS+1’) framework. Our workgroup created six modules: an introduction and one module focused on each of the Ms: (Mobility, Mentation, Medication, What Matters Most, and Multicomplexity). We created 60-minute, web-based modules to meet accreditation requirements of a broad spectrum of health professions while remaining feasible to complete in the context of other responsibilities. To enhance curriculum integration and adoption, we divided modules into 10–15-minute micro-learning segments. This presentation provides an overview of the process and challenges encountered in developing one such module for Multicomplexity. Providing trainees from varied backgrounds with an overview of Multicomplexity is daunting, given the breadth of topics encompassed and the differing levels of clinical knowledge among the targeted professions. A modified Delphi process helped narrow the focus to six topics: balancing treatment benefits and harms, engaging interprofessional teams, addressing communication challenges, frailty, care settings, and care transitions. Module materials were revised via an iterative process using input from colleagues and informal feedback from trainees. The final web-based training segments consisted of 508-compliant audio-visual presentations together with interactive cases, knowledge checks, and selected resources that ensured access to materials designed to support deeper learning and integration of geriatric best practices into care of older Veterans. Curriculum module development was a complex but worthwhile endeavor and required support from the national ILEAD office who provided assistance with 508 compliance, accreditation, and development of web-based trainings.

